# Exploring Community Readiness for Childhood Obesity Prevention: A Systematic Review of the Evidence

**DOI:** 10.5812/ijem-161812

**Published:** 2025-07-31

**Authors:** Mahdieh Niknam, Azin Zolfagharypoor, Mohammad Masih Mansouri-Tehrani, Parisa Amiri, Proushat Shirvani, Nasrin Omidvar

**Affiliations:** 1 Research Center for Social Determinants of Health, Research Institute for Metabolic and Obesity Disorders, Research Institute for Endocrine Sciences, Shahid Beheshti University of Medical Sciences, Tehran, Iran; 2 Department of Community Nutrition, National Nutrition and Food Technology Research Institute, Faculty of Nutrition Sciences and Food Technology, Shahid Beheshti University of Medical Sciences, Tehran, Iran

**Keywords:** Obesity Prevention, Community Readiness, Children, Community Readiness Model, Systematic Review

## Abstract

**Context:**

The community readiness (CR) level is a critical factor affecting the success and sustainability of prevention programs.

**Objectives:**

This study systematically reviews research utilizing the community readiness model (CRM) to assess CR for childhood obesity prevention programs, identifying readiness levels and summarizing their characteristics and outcomes.

**Methods:**

A comprehensive electronic and manual search was performed across multiple databases, including PubMed, Scopus, Web of Science, PsycINFO, Pub Psych, ERIC, ProQuest, and CINAHL, as well as two key journals: Preventing chronic disease and health education and behavior. The search adhered to predetermined inclusion and exclusion criteria. Studies evaluating CR for obesity prevention in children and adolescents aged 0 to 19 years were included, with no restrictions based on gender, race, ethnicity, or language. Three reviewers conducted data extraction and assessed the risk of bias using two appraisal forms from the Joanna Briggs Institute.

**Results:**

A total of 27 studies were included, with 24 conducted in high-income countries. Readiness levels across studies ranged from the first to the fifth stages. Communities generally reached the highest stage in the community effort dimension but were at the lowest stage regarding community climate and knowledge about the issue. The heterogeneity of the studies made it challenging to compare CR levels among different communities.

**Conclusions:**

This study provides an overview of CR levels and objectives of CR assessments, highlighting the significance of bottom-up interventions in childhood obesity prevention. It emphasizes the necessity of conducting context-specific readiness assessments before implementing interventions.

## 1. Context

Childhood obesity has rapidly increased worldwide during the last two decades (1). As a critical global health issue of the current century, it is demonstrated to be associated with adulthood obesity and various health and psycho-social complications (2). A recent study indicated that between 1990 and 2021, the combined prevalence of overweight and obesity among children and adolescents doubled, while the prevalence of obesity alone tripled (3). Given that childhood is a crucial time for lifestyle changes, promoting healthy habits in children is essential, as prevention is more cost-effective and effective than treatment (4, 5). Obesity is a complex issue with multiple contributing factors (6, 7), leading to a growing focus on community-based prevention strategies. Research highlights the importance of community involvement, particularly for children who often have limited control over unhealthy environments (8-11). However, successful prevention programs in one community may not replicate success in another if they lack the readiness to address the issue (12).

Community readiness (CR) refers to a community’s preparedness to accept and take action against an issue (13, 14). Readiness extends beyond initial adoption and implementation and reflects an organizational commitment, motivation, and capacity for change over time (15). Communities are at various stages of readiness for implementing plans, and quantifying readiness serves as a valuable tool for understanding the community’s current state to gain insights to define context-specific goals, strategies, and actions to motivate and shift the community to a higher level of readiness (12, 16).

Conducting a readiness assessment is essential for the effective implementation of preventive efforts, requiring a comprehensive approach tailored to the unique characteristics of the community (17). The community readiness model (CRM), developed by Oetting et al., is a widely used framework (16). Among previous studies that have specifically reviewed readiness, we found only three that investigated the literature for the concept, applications, and tools. In this regard, Castaneda et al. conducted a review study to integrate existing community and organizational readiness assessment models to determine the definition of readiness and assessment methods for innovative public health programs (17). Furthermore, a systematic review by Kostadinov et al. characterized the studies that applied CRM and demonstrated its application to various issues across social and health domains, including childhood obesity (18). Additionally, Schroder et al. used a scoping review approach to describe methods and modifications of the standard CRM protocol, and strategies to increase CR in the context of community-based childhood obesity prevention studies by examining 17 related studies (19). Despite the growing interest in childhood obesity prevention, no study has systematically reviewed the application of the CRM to quantify and classify readiness levels — both overall and by specific dimensions — in this context.

## 2. Objectives

Therefore, this study aims to systematically review existing research that utilizes the CRM to assess CR for childhood obesity prevention programs to identify levels of readiness and summarize their characteristics and outcomes. Providing detailed insights into CR levels is crucial for guiding decision-makers on the acceptance and implementation of childhood obesity prevention initiatives across diverse communities. These insights can pinpoint knowledge gaps to shape future research, encourage collaboration among stakeholders, and highlight the significance of addressing CR effectively.

## 3. Methods

### 3.1. Conceptual Framework

The CRM, developed from the trans-theoretical model by Oetting et al. (1995), quantifies CR through a mixed-method approach that incorporates both qualitative components and numerical scoring. Readiness is assessed by exploring and rating the opinions of key informants through in-depth interviews across six dimensions: (1) Existing prevention efforts; (2) community knowledge of these efforts; (3) leadership; (4) community climate; (5) community knowledge of the issue; and (6) available resources to support prevention efforts. The final CR score is calculated as the average of these six dimensions, following a predetermined protocol, and is rounded down to correspond to one of the nine stages of readiness (Appendix 1 in Supplementary File) (14, 16).

### 3.2. Data Sources and Search Strategy

The study was developed based on the Preferred Reporting Items for Systematic Reviews and Meta-Analyses (PRISMA) statement (20). The study protocol is registered with the International Prospective Register of Systematic Reviews (PROSPERO; CRD42018107831). A thorough electronic and manual search was conducted across multiple databases, including PubMed, Scopus, Web of Science, PsycINFO, PubPsych, ERIC, ProQuest, and CINAHL, as well as two key journals: Preventing chronic disease and health education and behavior. The search also included gray literature, such as conference papers and dissertations, using the same databases. To ensure comprehensive coverage, the references of identified studies, related narratives, and systematic reviews were examined. Appropriate keywords were developed using Medical Subject Headings (MeSH) and free text terms, with search terms including variations of "readiness" and "community" concepts (Appendix 2 in Supplementary File). A primary search syntax was initially crafted for PubMed, which was then refined based on the number needed to read (NNR) for efficiency before adapting it for other databases. Notably, the search time interval was extended from the originally registered period of January 1, 2000, to December 31, 2024.

### 3.3. Study Selection

In this study, community-based studies are defined as research focusing on groups or specific geographic communities rather than individuals. Articles and gray literature were included if they were published between 2000 and 2024, evaluated CR for preventing obesity in children and adolescents aged 0 to 19 years, and imposed no restrictions based on gender, race, ethnicity, or language. Studies on obesity management and those assessing CR in individuals over 19 years of age were excluded. After importing references into EndNote X7 and removing duplicates, three reviewers screened studies based on titles and abstracts, followed by full-text assessments, ultimately categorizing articles into three subgroups: Included, excluded, and borderline.

### 3.4. Data Extraction and Risk of Bias Assessment

Two reviewers developed the data extraction form. After piloting the form, they independently filled it out by extracting data from the included articles, which encompassed study design, participants, communities, and outcomes. Following data extraction, two appraisal assessment forms from the Joanna Briggs Institute (21) were combined to create a 12-item checklist for evaluating analytical cross-sectional studies (n = 8) and qualitative research studies (n = 10). This checklist included ten questions adapted from the analytical studies, with two modified into separate questions, and five questions from the qualitative studies. The final risk of bias assessment covered key areas such as inclusion criteria for key informants, study subject and setting descriptions, measurement criteria, validity and reliability of outcomes, availability of CR data, methodological congruence, participant representation, ethical approval, and conclusions drawn from data analysis. Two independent reviewers assessed the selected studies, categorizing them as good (11, 12), satisfactory (10), or unsatisfactory (≤ 9).

### 3.5. Dealing with Missing Data and Disagreements

The authors attempted to contact the study authors for unpublished data or inaccessible full texts at least three times, spaced 10 days apart. Disagreements between reviewers during study selection, data extraction, and bias assessment were resolved through discussion or referral to corresponding reviewers for consensus. Inter-rater agreement was measured using the KAPPA Index (22).

## 4. Results

### 4.1. Included Studies

**Figure 1.**
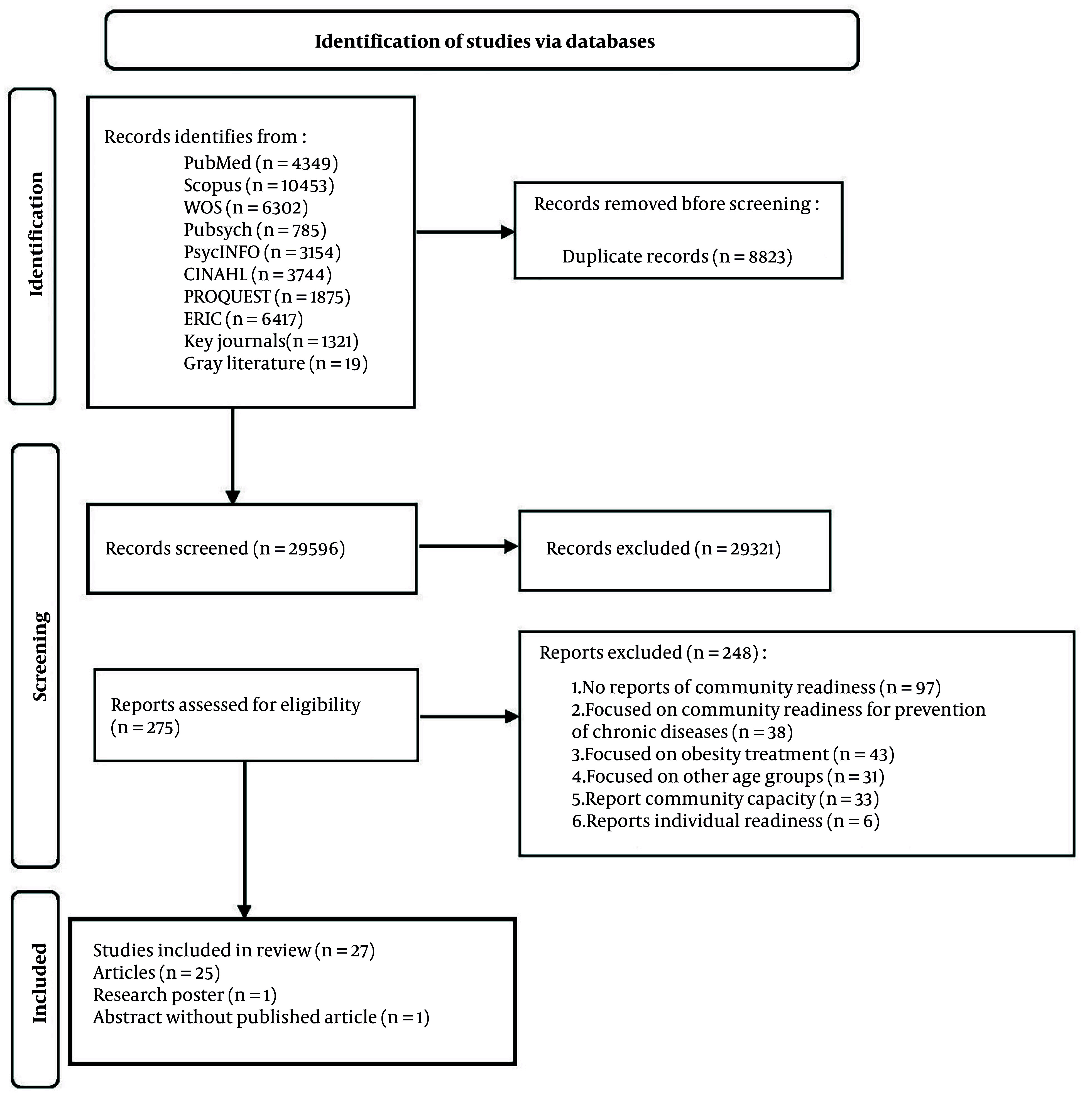
Preferred Reporting Items for Systematic Reviews and Meta-Analyses (PRISMA) flow diagram

The study selection process is summarized in Figure 1. The search of major databases and key journals provided 38,419 records. After removing 8,823 duplicates, 29,596 underwent titles and abstract screening to identify potentially eligible studies. Subsequently, 29,321 irrelevant studies were excluded, and 275 relevant ones were included for full-text review. Finally, up to the end of December 2024, 27 distinct studies, including 25 articles, one research poster (23), and one abstract (24), were included in our systematic review. Intra-rater reliability among independent reviewers for 275 articles indicated an acceptable level of agreement (KAPPA = 0.71). The study's exclusion was primarily due to the lack of qualitative or quantitative reports on CR levels.

### 4.2. Study Characteristics

The characteristics of the included studies are presented in Table 1. The publication dates of the identified studies ranged from 2007 to 2024 and were conducted in nine countries. Except for one (25), all included studies were in English. The first study was conducted in the USA (26). In total, 24 out of 27 (80%) studies were conducted in high-income countries, including the USA (14 studies) (23, 24, 27-37), Australia (5 studies) (38-42), the UK (2 studies) (43, 44), Canada (45), and Germany (46). The remaining four reported the CR from upper- to lower-middle-income countries, including Costa Rica (1 study) (25), South Africa (1 study) (47), and Iran (2 studies) (48, 50). Most studies were conducted in urban areas, while others occurred in rural (23, 26, 27, 31, 32, 34, 42, 45) or both urban and rural areas (19, 36, 39, 51).

**Table 1.** Characteristics of the Included Studies in the Systematic Review (N = 27)

First Author, Publication, Year	Study Site	Country’s Economic Status	Study design					Number of Communities	Number of Key Informants	Duration of Data Collection (mo)	Key Findings
			Study Type	Urban/Rural Area	Target Group Age (y)	Sex	Data Collection Method				
**Findholt, 2007 (** 26 **)**	Union County, Oregon, USA	High income	Qualitative study	Rural	Children, ND	ND	In-person interview	1	15	2	The final CR Score was 1.6, which, when rounded down, corresponded to the first stage of readiness: No awareness.
**Sliwa, 2011 (** 37 **)**	Eight states, USA	High income	Mixed-method study	Urban	Children ND	ND	In-person interview	10	40	0.5	The mean readiness score of 10 communities was at the fourth stage, preplanning.
**Frerichs, 2012 (** 35 **)**	Latino community, Nebraska, USA	High income	Mixed-method study	Urban	Youth ND	ND	In-person interview	1	18	4	South Omaha community was at a low stage of readiness to address childhood obesity (stage 3: Vague awareness).
**Ehlers, 2013 (** 36 **)**	Midwestern Metropolitan area, USA	High income	Qualitative study	Both	Children 8 - 13	ND	In-person interview	17	101	ND	The mean readiness score was corresponds with a "vague awareness" level of readiness.
**Kesten, 2013 (** 43 **)**	Charlwood Borough, UK	High income	Qualitative study	Both	Pre-adolescence 7 - 11	Girls	In-person interview	6	33	10	The CRM assessment suggests that this community is in the ‘Initiation’ and ‘Preparation’ stage for physical activity and healthy eating and drinking, respectively.
**Alcócer Alfaro, 2013 (** 25 **)**	Concepcion de la Union, Costa Rica	Upper-middle income	Mixed-method study	Urban	Children ND	ND	In-person interview	1	15	7	The CR was found to be in a stage of denial, which means very little recognition that childhood obesity is a community problem.

Abbreviations: ND, not determined; CR, community readiness; CRM, community readiness model.

### 4.3. Communities, Key Informants, and Conceptual Framework

Definitions of community varied across the included studies. In most cases, the community was defined exclusively based on geographical boundaries, e.g., urban communities (n = 6) (34, 36, 37, 40, 42, 43), counties (n = 5) (26, 27, 29, 30, 34), rural communities (n = 6) (23, 31, 32, 34, 42, 45), and suburbs (n = 1) (39). Other approaches used for defining the communities were based on institutions and shared interests, e.g., schools (n = 3) (44, 47, 48), churches (n = 1) (47), and municipalities (46). The number of communities included in each study varied between one and 42 (9.1 ± 8.8; median = 6); five out of 27 studies focused on one community (25, 26, 28, 31, 35) and eight studies had ≤ 10 communities (35, 39-41, 44, 46, 51, 52). Only 12 studies reported the duration of data collection, which ranged from 0.5 to 10 months (4.6 ± 2.8; median = 3.0) (25-27, 35, 37, 44-48, 50, 51).

The number of key informants interviewed varied between six (44) and 114 (40) (47.9 ± 32.5; median = 40) and was unclear in three studies (23, 32, 34). In addition, the key informants had different roles with various community connections due to differences in the purpose and setting of the study. All studies used the snowball sampling approach to select the key respondents, except for Kesten et al. (43) and Harris et al. (27), which used focus group discussions (FGDs) and computer-generated random sampling to select key informants, respectively.

All included studies used CRM as the conceptual framework. Except for 13 studies that deleted (32, 36, 38, 40) or added some questions (25, 26, 34, 37, 41, 42, 45, 48, 50) to modify the CRM interview questions, other studies employed CRM following the standard protocol by asking 36 adaptable questions to accommodate various issues or contexts. In addition to the number of questions, other modifications were made to the standard CRM protocol, such as adjusting the number of key respondents. While the protocol recommends using four to six key informants per community, 23 studies included more key informants (34-40, 43-53), and three studies utilized fewer than the recommended number (24, 40, 44). The CRM modifications were also identified regarding the data collection method. Despite the protocol’s recommendation for utilizing structured in-person or phone-guided interviews, two studies deviated from this approach and employed an online survey (24, 39). Six studies employed diverse data collection methods — such as surveys, observations, FGDs, photo mapping (PM), and community conversations (CCs) — either exclusively or alongside structured interviews (23, 31-33, 39, 47).

### 4.4. Main Findings

The overall readiness stages reported by the 27 included studies ranged from 1 to 5 (Appendix 3 in Supplementary File). Four studies reported only the total readiness stages (23, 25, 26, 40), one study reported CR only for the leadership dimension without providing the total (39), and others (22 of 27 studies) reported both total and dimension-specific readiness stages. Eleven studies reported the stage of CR based on the children’s age (23, 24, 28, 36, 39-41, 43, 45, 48, 50), and three presented the readiness based on the children’s gender (43, 48, 49). Although comparing readiness across different communities was difficult due to significant variations in definitions of "community" and differences in the characteristics of children and key informants involved in the studies, as well as the methodological diversity.

The overall CR stages from the included studies are presented as follows.

#### 4.4.1. Stage 1: No Awareness

In a study conducted in a rural community in Union County, USA, the overall CR was classified at the first stage of readiness. This was the first application of the CRM to childhood obesity prevention (26).

#### 4.4.2. Stage 2: Denial/Resistance

Four studies were identified as being at the second stage of CR. These studies were conducted in both rural and urban areas, encompassing diverse locations such as churches and one underserved urban community in South Africa and Costa Rica, which are classified as upper-middle-income countries (25, 47). Additionally, studies were conducted in schools and rural counties in Australia and the USA, considered high-income countries (27, 41). The number of interviewed key informants varied from 15 to 54.

#### 4.4.3. Stage 3: Vague Awareness

Most communities (12 out of 27) demonstrated the third stage of readiness, referred to as vague awareness (23, 28, 31, 32, 34-36, 40, 42, 44-46). These studies were exclusively conducted in high-income countries (eight in the USA, one in Australia, one in the UK, one in Canada, and one in Germany). Moreover, these studies encompassed diverse locations, such as rural, urban, or a combination of both, and were conducted in various settings, including urban communities, rural communities, counties, schools, and municipalities. The number of key informants interviewed in these studies ranged from 6 to 114. One study did not report any dimensions of readiness (40). Notably, the HEAL MAPPS study, a sizeable grant-funded child obesity prevention study conducted in six states of the USA involving 21 rural communities, demonstrated a total readiness level corresponding to vague awareness. However, based on the information available on oregonstate.edu, the CR stage of each rural community ranged from 2 to 4 (Appendix 3 in Supplementary File) (52).

#### 4.4.4. Stage 4: Preplanning

The overall CR of the seven studies corresponded to the fourth stage (preplanning). These studies were conducted in urban communities in both high-income (29, 30, 37, 38, 45) and low-income (48, 50) countries, where 20 to 95 key informants participated. As illustrated in Appendix 3 in Supplementary File, there was notable unity among the dimension-specific readiness stages, and the majority were at the preplanning stage. The “community effort” and “community climate” received the highest and lowest stages, respectively. Only one study reported the CR separately for girls and boys in school-age children, which showed that the readiness of target communities in girls’ schools was higher than in boys’ schools due to allocating more resources, having more involved leaders, and community motivation (48).

#### 4.4.5. Stage 5: Preparation

Only two studies, conducted by Kesten et al. (43) and He et al. (45), reported an overall CR at the fifth stage, indicating active planning by community leaders to secure resources for obesity prevention. These pioneering studies in the UK and Canada focused on different age groups: Kesten et al. on pre-adolescent girls (ages 7 - 11) and He et al. on children up to age 8. Kesten's study uniquely assessed CR for physical activity and healthy eating separately, finding higher readiness for physical activity (stage 6) compared to healthy eating (stage 5). The readiness dimensions varied, with community knowledge and climate at the preparation stage, while leadership was at the initiation stage and community efforts at stabilization (stage 7) (43, 45). He et al.'s study in Rockwood, Canada, showed varied readiness across dimensions, with community efforts at the initiation stage, community knowledge and climate at preparation, and leadership and issue knowledge at the preplanning stage (stage 4) (45).

It is important to note that the total CR was unclear in one particular study. This study, conducted by Fialkowski et al. in 2013, was only available in abstract form without a published article. The study evaluated the CR of 20 urban communities across five jurisdictions in the USA. While the CR level for each jurisdiction was presented, ranging from vague awareness in Alaska to initiation in American Samoa, the overall readiness status of all five jurisdictions was undetermined (24).

### 4.5. Community Readiness by Dimensions

The CR based on each dimension of CRM is presented in Appendix 3 in Supplementary File.

#### 4.5.1. Community Efforts and Community Knowledge of the Efforts

All of the studies achieved the highest stage of readiness for the "community effort" dimension. The readiness stages for this dimension ranged from stages 4 to 7, except for the study by Pradeilles et al. in 2016, which reported a readiness stage of 2 (47). In contrast, the "community knowledge of the efforts" dimension received lower readiness levels, ranging from stage 1 to 5. Interestingly, three studies reported the readiness stage for only five dimensions instead of six. These studies combined the "community efforts" and "community knowledge of the efforts" into one dimension and reported the readiness stage exclusively for the "community knowledge of the efforts" dimension (28, 34, 42).

#### 4.5.2. Community Climate and Community Knowledge of the Issue

Despite the high stage of readiness for the "community efforts" dimension, all studies reported the lowest stage of readiness for the "community climate" and "community knowledge of the issue" dimensions, which are related to community attitude and awareness. This may indicate a top-down approach to addressing childhood obesity. Although the readiness for these dimensions ranged from stages 2 to 4 (except for Harris et al. in 2019 (27), Kesten et al. in 2013 (43), and He et al. in 2023 (45), they were predominantly situated at the second and third stages of readiness.

#### 4.5.3. Leadership and Resources

The readiness for the “leadership” dimension was at the second, third, and fourth stages in most of the studies (except Heath et al. 2020 (29)/stage 5 and Kesten et al. 2013 (43)/stage 6). Furthermore, one study solely reported the community’s perception of the “leadership” dimension, and the result showed that readiness corresponded to vague awareness (39). The scores for “resources” placed this dimension in the second, third, and fourth stages (except Heath et al. (29), Sheldon et al. (30), and Kesten et al. (43)/both in stage 5). Hence, these dimensions were mainly placed between the highest and lowest stages among the CR dimension-specific stages.

#### 4.5.4. Risk of Bias in Included Studies

**Figure 2.**
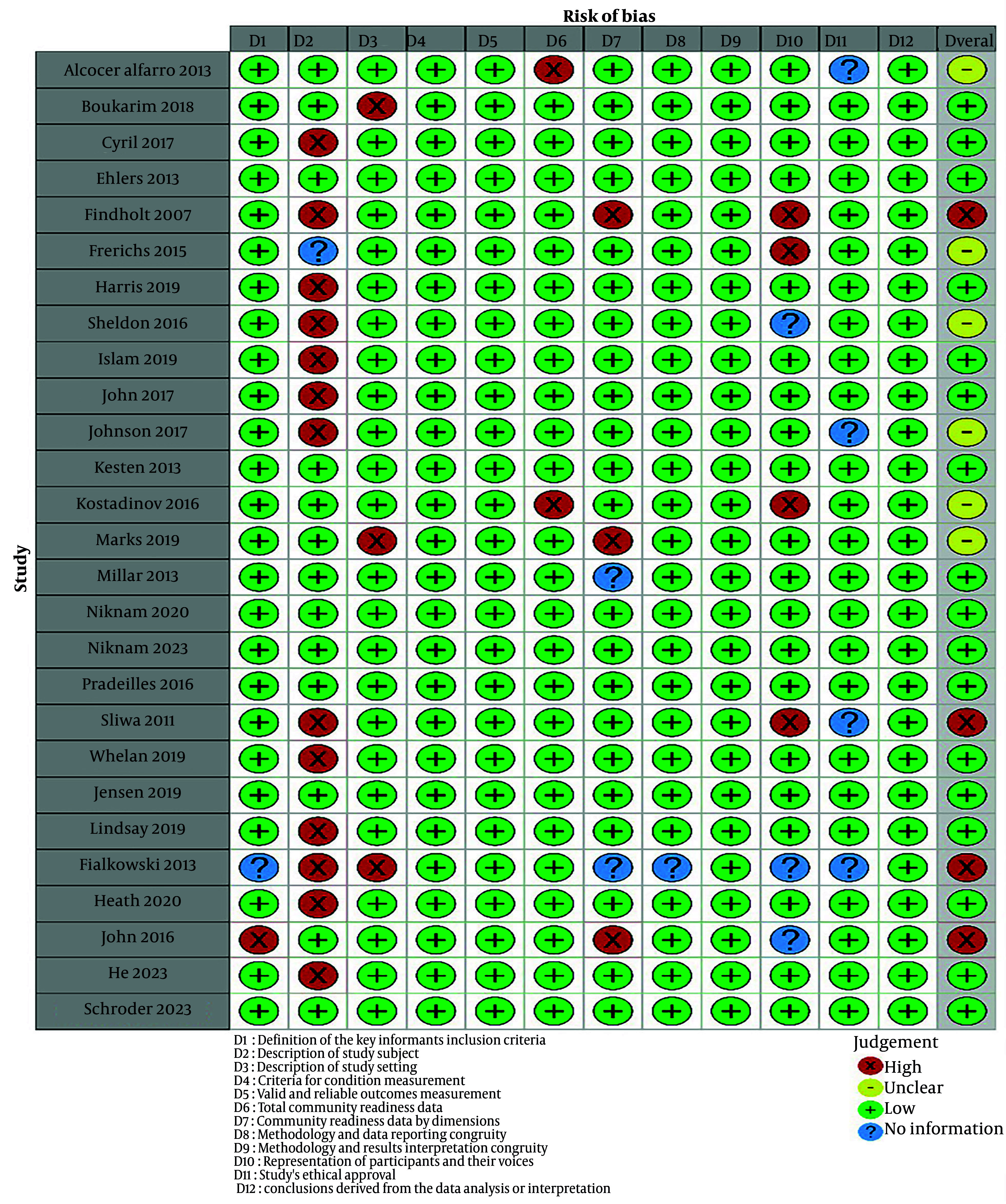
Risk of bias assessment for individual studies (+ low risk of bias; - high risk of bias; ? unclear: Insufficient information about each item to permit judgment of low-risk or high-risk) (23-49).

**Figure 3.**
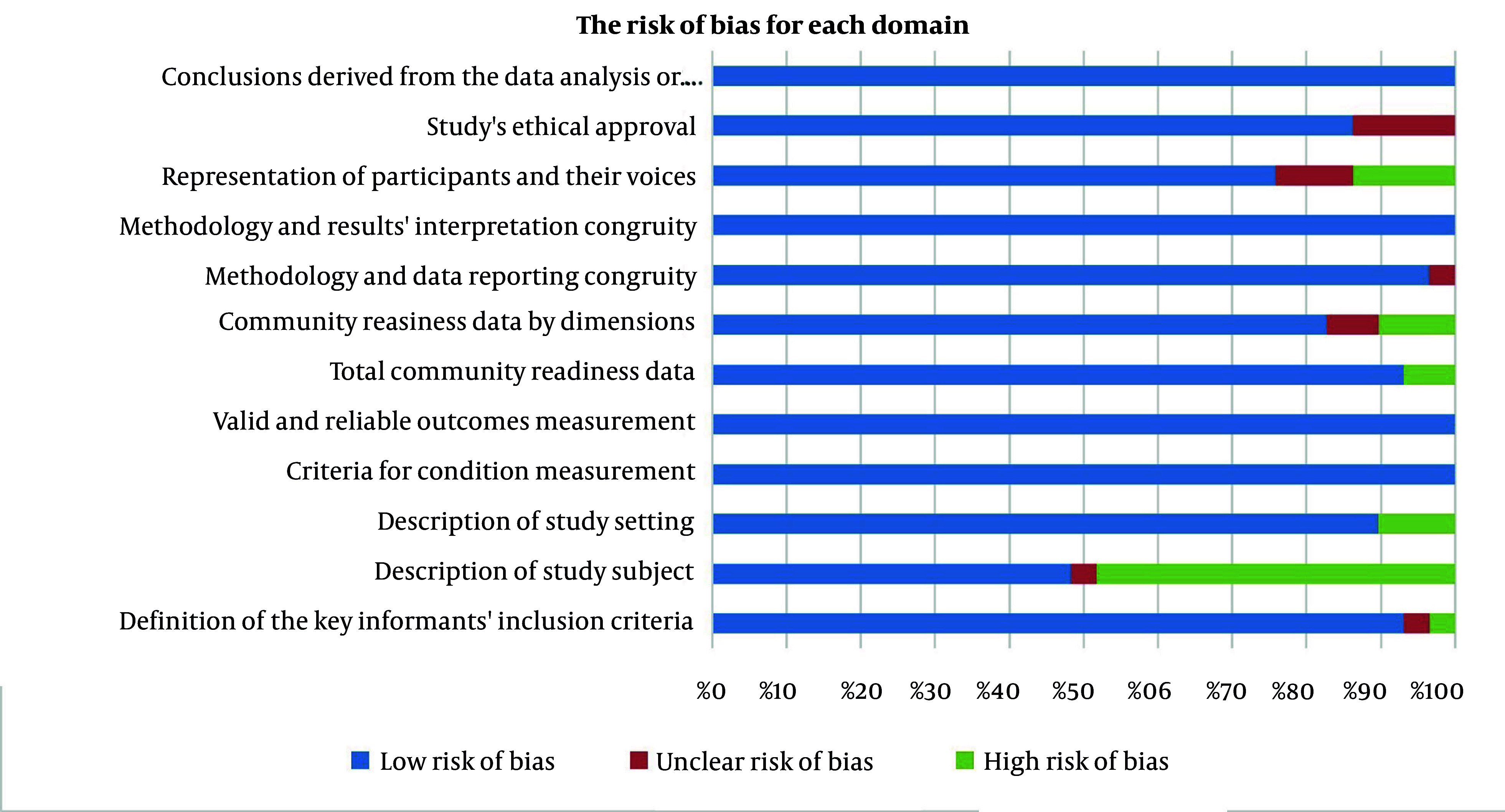
The risk of bias for each domain

The risk of bias for each study is demonstrated in Figure 2 and as a percentage for each domain in Figure 3. In all studies, 7 out of 27 studies (31, 36, 43, 46-48, 50) were at low risk of bias for all domains of quality assessment. In total, all domain-related bias was mainly low; however, the description of the study subject had the highest risk of bias for 50% of studies. Reporting biases associated with the description of the study setting, except for 10% of the studies including Marks et al. (40), Boukarim (28), and Fialkowski et al. (24), and the total CR data, except for about 5% of studies including Kostadinov et al. (39) and Alcocer Alfaro (25), were low. There was an unclear risk in ethical approval reporting for 15% of studies (24, 25, 34, 37). Additionally, three studies on the representation of participants and their voices (23, 24, 30), two on the CR data by dimensions (24, 41), and one on the description of the study subject (35) presented an unclear risk of bias due to inadequate data. Studies with a high risk of bias were not excluded from the study.

## 5. Discussion

The systematic review identified 27 studies on CR for childhood obesity prevention, primarily conducted in urban, high-income areas. The findings indicate low to mid-range levels of preparedness, with none demonstrating high overall preparedness. Communities demonstrated the highest readiness in "community effort", while "community climate" and "community knowledge of the issue" had the lowest levels. Additionally, comparing readiness across different communities was difficult due to significant variations in definitions of "community" and differences in the characteristics of children and key informants involved in the studies.

This study revealed that the readiness of all identified studies fell within the first to fifth stages, indicating low to mid-range preparedness, with no communities exhibiting a high level of readiness. Most studies corresponded to the third and fourth stages of readiness. The third stage reflects vague awareness, signifying only a general local concern about the issue. In contrast, the fourth stage indicates more than just concern, revealing limited and unsecured resources, along with unsustainable and unfocused efforts toward addressing childhood obesity (18).

All studies that reached the third stage of readiness were exclusively conducted in high-income countries (23, 28, 31, 32, 34-36, 40, 42, 44-46). However, considering the alarming epidemiological data on childhood obesity in Western countries (53) and the publication dates of these studies, which span from 2012 to 2024, the observed low level of readiness is concerning and prompts further reflection. Notably, except for one study (45), all studies that reached the fourth stage of readiness were focused on urban communities (29, 30, 37, 38, 48, 50). However, drawing meaningful interpretations is challenging because the readiness levels of other included studies targeting urban communities varied between the second and fourth stages.

This study found that communities primarily achieved the highest readiness stage in the "community effort" dimension, ranging from the fifth to seventh stages. These efforts predominantly focused on promoting healthy eating and physical activity. However, some communities exceeded these initiatives by emphasizing the generation of social movements, investing in people, establishing community coalitions, and fostering cross-sectoral collaboration, thereby attaining a high level of readiness in the "community effort" dimension (29, 30, 35, 37, 38, 43-45).

Additionally, our findings revealed that the "knowledge of the effort" dimension predominantly fell within the lower stages compared to the "community efforts" dimension. This highlights a gap between the implemented efforts and the understanding among community residents, indicating difficulties, a lack of interest, and insufficient resources dedicated to increasing awareness and engagement within the community (30, 37, 38).

This study indicates that the "community climate" and "community knowledge of the issue" dimensions received the lowest readiness stages. Previous studies have established a close association between these two dimensions, as the community’s perception and understanding of a problem are crucial alongside awareness (17). The low levels of readiness for these dimensions indicate a misunderstanding about the prevention concept (stage 2) (27, 28, 34-36), lack of information, limited resources, and existing more pressing problems and issues than childhood obesity in the public healthcare system (stage 3) (32, 34, 37, 42), and lack of statistics and existing misconceptions about childhood obesity (stage 4) (48, 49).

The current study revealed that the "leadership" and "resources" dimensions predominantly fell between the second and fourth readiness stages, indicating a range between the highest and lowest levels of readiness across studies. These findings suggest that even at the most advanced readiness stage, resources remain limited, and leaders committed to addressing childhood obesity may show reduced motivation to pursue further efforts (18). This highlights challenges within the "leadership" dimension, indicating that while some engagement exists, constraints regarding available resources and sustained commitment persist in tackling childhood obesity.

The current study indicated that most included studies were conducted in urban areas and high-income countries, primarily the USA and Australia. Similar to this finding, a previous systematic review showed that the studies predominantly applied CRM in the USA, where the model was initially developed (18). Furthermore, childhood obesity first emerged as a significant public health challenge in developed countries (1). Consequently, these countries recognized the importance of community-based settings in obesity management early on and turned to community strategies and social supports for effective prevention and intervention measures (11).

Furthermore, the focus on urban rather than rural areas in these studies may be due to smaller populations, larger geographic areas, limited resources, and difficulties in attracting specialists to rural settings (18). Defining an area as urban or rural is complex and becomes even more complicated when the target community involves a large population; hence, our study classified the studies into one or both of these delineations based on the study's report. The higher number of studies conducted in urban areas compared to rural areas is not surprising. It can be attributed to factors such as smaller population sizes, larger geographic areas, the scarcity of resources, and the challenges associated with attracting specialists in rural settings (18).

However, drawing meaningful interpretations regarding the importance of study site, urban vs. rural, and high-income vs. low/middle-income countries is challenging due to the variation in their readiness levels and methodology. In this regard, adapting the CRM specifically for low-resource settings involves simplifying tools, fostering community-led prioritization, and enhancing local capacity through concise, culturally relevant surveys and stakeholder engagement (49). Training local facilitators and utilizing existing community resources are essential for sustainability (19). Additionally, the use of digital tools, particularly mobile surveys, enhances data collection by improving accessibility, engagement, and data quality. These tools facilitate real-time data collection, increase response rates, reduce costs, and minimize errors, ultimately empowering communities to take ownership of their health initiatives and ensuring that assessments remain relevant to local needs (15, 48).

The current study encountered challenges in comparing readiness levels among different societies due to significant heterogeneity in the characteristics of the studied communities, children, and key informants. The concept of CR is context-dependent, and therefore, establishing a clear definition of the community is crucial before applying the concept (18). However, the definition and boundaries of communities varied across the identified studies, leading to ambiguity in how areas were classified as rural or urban and how these classifications were consistently applied or altered in different investigations. This inconsistency hampers the ability to compare readiness across studies.

Furthermore, despite a growing body of literature highlighting the importance of children's age and gender in weight gain, as well as the influence of leaders' attitudes and community dynamics, these factors were not consistently addressed in the reviewed studies (51). Additionally, our findings revealed substantial variation in the number and roles of key informants across studies, primarily due to differences in the study’s purpose and setting. As a result, comparing readiness data based on key informants became impossible (16).

The current study is the first systematic review examining CR levels for childhood obesity prevention programs. An extensive search of multiple databases, key journals, and grey literature adds valuable insights to the limited evidence on CR in this context. However, several limitations should be noted. First, CR data availability was restricted in some studies, and despite repeated attempts to contact authors, necessary information could not be obtained. Second, the heterogeneity among the included studies — regarding settings, communities, and participants — complicated the differentiation of CR levels based on specific characteristics, potentially introducing confounding variables and hindering direct comparisons. Additionally, changes in CR over time were not explored, as the focus was solely on cross-sectional assessments at the baseline of the studies. Lastly, the potential impact of publication bias on the distribution of CR stages should be taken into account during interpretation.

### 5.1. Conclusions

The studies included in the review consistently showed low (stages 1 to 3) to moderate (stages 4 to 5) readiness levels among communities for developing and implementing childhood obesity prevention programs. These findings underscore the need to enhance readiness, particularly in areas with low levels. The highest readiness was observed in the "community effort" dimension, while the "community climate" and "community knowledge of the issue" dimensions exhibited the lowest levels. This disparity indicates a top-down approach to addressing childhood obesity, suggesting that existing efforts are strong at the planning and decision-making levels in most communities studied. However, it highlights the necessity for greater emphasis on bottom-up interventions that prioritize community awareness, involvement, and the creation of a supportive environment. This finding further underscores the significance of CR assessments that focus on capacity-building in the dimensions of "community climate" and "knowledge" for future interventions. Additionally, it highlights the need for standardized reporting of CRM adaptations to enhance cross-study comparisons.

ijem-23-3-161812.pdf

## Data Availability

The dataset presented in the study is available on request from the corresponding author during submission or after publication. The data are not publicly available due to the substantial time, effort, and resources invested in their collection. However, the data can be shared with the journal or researchers upon reasonable request.
